# Intracranial phosphaturic mesenchymal tumor mimicking meningioma and causing tumor-induced osteomalacia: a case report

**DOI:** 10.1093/jbmrpl/ziag156

**Published:** 2026-09-17

**Authors:** Samaneh Rabiei, Daniel Collins, Paul Schwartz, Antonella Pallante, Xianyuan Song, Vitaly Kantorovich

**Affiliations:** Department of Medicine, Division of Endocrinology, Hartford Hospital, Hartford, CT 06102, United States; Department of Radiology, Hartford Hospital, Hartford, CT 06102, United States; Department of Neurosurgery, Hartford Hospital, Hartford, CT 06102, United States; Department of Pathology, Hartford Hospital, Hartford, CT 06102, United States; Department of Pathology, Hartford Hospital, Hartford, CT 06102, United States; Department of Medicine, Division of Endocrinology, Hartford Hospital, Hartford, CT 06102, United States

**Keywords:** tumor-induced osteomalacia, phosphaturic mesenchymal tumor, FGF-23, hypophosphatemia, meningioma

## Abstract

A 32-yr-old woman presented with a 2-year history of progressive bone pain, proximal muscle weakness, worsening ambulation, and bilateral hip insufficiency fractures without preceding trauma. Laboratory evaluation revealed severe hypophosphatemia (1.1 mg/dL), elevated bone-specific alkaline phosphatase, progressive secondary hyperparathyroidism, and inappropriately elevated circulating FGF-23, with fasting paired serum and urine studies confirming renal phosphate wasting (tubular maximum phosphate reabsorption per glomerular filtration rate 1.88 mg/dL; fractional excretion of phosphate 14.6%), consistent with tumor-induced osteomalacia (TIO). Functional imaging with ^68^Ga-DOTATATE PET/CT demonstrated intense tracer uptake in a small anterior cranial lesion. Brain MRI revealed a dural-based olfactory groove lesion interpreted radiographically as a meningioma. Selective venous sampling demonstrated a cranial-to-peripheral gradient in intact FGF-23 consistent with drainage from the anterior cranial fossa. Surgical resection produced postoperative normalization of FGF-23 and rapid correction of hypophosphatemia. Histology showed meningioma-like architecture with whorls and psammoma bodies, but expanded molecular testing did not identify canonical meningioma-associated alterations, and DNA methylation profiling classified the lesion as a mesenchymal neoplasm not elsewhere classified and fusion negative. FGF-23 in situ hybridization was positive, and the final surgical pathology diagnosis was mesenchymal tumor favoring phosphaturic mesenchymal tumor over meningioma. This case illustrates how a mesenchymal tumor arising in the meninges can mimic meningioma radiographically and histologically while producing the endocrine phenotype of TIO.

## Graphical Abstract

**Graphical Abstract:**
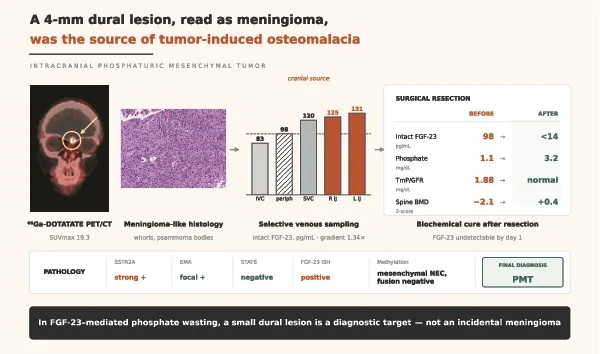

## Introduction

Tumor-induced osteomalacia (TIO) is an acquired paraneoplastic syndrome caused by excess secretion of FGF-23, which reduces renal tubular phosphate reabsorption and suppresses 1,25-dihydroxyvitamin D synthesis, leading to chronic hypophosphatemia and defective skeletal mineralization. In most cases, the responsible lesion is a phosphaturic mesenchymal tumor (PMT), but localization is often delayed because these tumors are typically small and can arise in diverse anatomic sites. The diagnostic challenge becomes greater when tumor localization implicates an intracranial lesion, particularly one that appears radiographically consistent with meningioma, a common and often incidental dural-based tumor.

In this case, the central clinical problem was determining whether a small olfactory groove lesion interpreted as a meningioma represented incidental pathology or the source of the patient’s FGF-23–mediated phosphate wasting.

## Case presentation

A 32-yr-old woman presented with a 2-year history of progressive bilateral hip pain, diffuse bone pain, and worsening difficulty with ambulation, eventually requiring a cane. She denied preceding trauma, falls, or family history of metabolic bone disease. At presentation she was taking no prescription medications and no over-the-counter supplements, including no vitamin D, calcium, phosphate, or phosphate-binding antacids, and she had no history of exposure to intravenous iron formulations, tenofovir, or other agents associated with hypophosphatemia or proximal tubular dysfunction. Her symptoms progressively limited mobility and daily activities.

MRI of the hips demonstrated chronic right femoral neck and left intertrochanteric insufficiency fractures, rather than Looser–Milkman pseudofractures, with signs of healing. DXA revealed low BMD, with a LS BMD of 0.98 g/cm^2^ (Z-score −2.1) and a left FN BMD of 0.39 g/cm^2^ (Z-score −5.3). Because of worsening pain and functional decline, she was hospitalized and underwent surgical fixation of bilateral hip insufficiency fractures.

Laboratory evaluation revealed severe hypophosphatemia at 1.1 mg/dL (reference range: 2.5-4.5 mg/dL), normal serum calcium, suboptimal 25OHD at 24 ng/mL, and elevated bone-specific alkaline phosphatase at 61.3 μg/L (reference range: 5.3-19.5 μg/L). Parathyroid hormone was 50 pg/mL at presentation and rose to a peak of 163 pg/mL over the subsequent 8 mo (reference range: 16-77 pg/mL), consistent with progressive secondary hyperparathyroidism.

Renal phosphate handling was assessed on a fasting second-morning-void urine specimen paired with a simultaneous fasting serum sample, both obtained while off phosphate supplementation. These demonstrated renal phosphate wasting, with a tubular reabsorption of phosphate of 85.4%, a fractional excretion of phosphate of 14.6%, and an inappropriately low tubular maximum phosphate reabsorption per glomerular filtration rate (1.88 mg/dL; reference range: 2.5-4.2 mg/dL).^1^

Circulating FGF-23 was inappropriately elevated, supporting a diagnosis of FGF-23–dependent hypophosphatemia. Two different FGF-23 assays were used over the course of the evaluation and their values are not interchangeable. Initial screening used a C-terminal FGF-23 assay (Pan Laboratories; adult reference range: <180 RU/mL), which was elevated at 284 RU/mL. All subsequent measurements—diagnostic confirmation, every selective venous sampling specimen, and all postoperative surveillance—used the intact FGF-23 assay at Mayo Clinic Laboratories (test IFG23; reference value 59 pg/mL or below), which detects only the full-length, biologically active hormone. The intact assay was performed on a MedFrontier platform throughout, although the laboratory transitioned format during follow-up: specimens analyzed at the time of diagnosis and in the immediate postoperative period used an enzyme-linked immunosorbent assay, and the specimen obtained 10.5 mo postoperatively was analyzed by a chemiluminescence-based quantitative sandwich immunoassay. Both are intact-specific methods, and values from the 2 FGF-23 assays are reported separately with their respective units throughout (Table 1).

**Table 1:** Biochemical parameters before and after resection.

**Parameter (ref. range)**	**Baseline**	**Pre-Op**	**POD 1**	**10 d**	**1 mo**	**4 mo**	**10.5 mo**	**17.5 mo**
**Phosphorus (2.5-4.5 mg/dL)**	1.1	1.2	2.8	4.1	4.6	3.6	3.8	3.2
**Calcium, mg/dL (8.6-10.2)**	9.3	9.0	—	9.3	10.0	9.7	9.7	9.8
**PTH, intact, pg/mL (16-77)**	50	163	—	75	—	37	44	—
**25-hydroxyvitamin D, ng/mL (30-100)**	24	—	—	28	44	42	33	51
**1,25-dihydroxyvitamin D, pg/mL (18-72)**	29	—	—	—	218	129	142	—
**Total alkaline phosphatase, U/L (31-125)**	235	—	—	—	149	133	—	72
**Bone-specific ALP, μg/L (5.3-19.5)**	61.3	—	—	—	43.3	—	—	—
**Intact FGF-23, pg/mL (≤59)^a^**	—	98	<14	—	—	—	18	—
**C-terminal FGF-23, RU/mL (<180)^b^**	284	—	—	123	—	—	—	—
**Urine phosphorus, random, mg/dL**	27	—	—	95	—	31	<10	<10
**Urine creatinine, random, mg/dL**	37	—	—	64	—	—	36	8
**TRP, %**	85.4	—	—	—	—	—	95.8	—
**TmP/GFR, mg/dL (2.5-4.2)**	1.88	—	—	—	—	—	—	—

Biochemical parameters before and after resection of the olfactory groove lesion; intervals are relative to the date of surgery.

Urine phosphorus below 10 mg/dL is below the laboratory reporting limit; paired random urine creatinine is shown to allow assessment of specimen concentration. The preoperative parathyroid hormone value is the peak recorded during the preoperative period.

Abbreviations: ALP, alkaline phosphatase; POD, postoperative day; TmP/GFR, tubular maximum phosphate reabsorption per glomerular filtration rate; TRP, tubular reabsorption of phosphate.

a Intact FGF-23, serum, Mayo Clinic Laboratories; the preoperative value is the peripheral (right common femoral) specimen obtained at selective venous sampling.

b C-terminal FGF-23, a different analyte reported in different units; the 2 FGF-23 rows are not directly comparable.

### Differential diagnosis and investigations

The combination of adult-onset hypophosphatemia with renal phosphate wasting and an inappropriately elevated intact FGF-23 established an FGF-23–dependent process and narrowed the differential to acquired and hereditary causes. Inherited FGF-23–mediated disorders—X-linked hypophosphatemia and autosomal dominant and autosomal recessive hypophosphatemic rickets—were considered unlikely given the adult onset, the absence of childhood rickets, short stature, or lower-limb deformity, normal growth and dentition, and no family history of metabolic bone disease. Tumor-induced osteomalacia (TIO) was therefore suspected.

Functional imaging with ^68^Ga-DOTATATE PET/CT demonstrated intense radiotracer uptake (maximum standardized uptake value: 19.3) along the midline at the junction of the ethmoid air cells and adjacent nasal cavity, with mild uptake within the axial skeleton and femora (Figure 1). Brain MRI revealed focal dural thickening along the left olfactory groove and inferior left frontal lobe, measuring 4 mm in thickness, radiographically suggestive of a meningioma. On co-registered review, the focus of intense radiotracer uptake corresponded to the olfactory groove lesion; no other site of abnormal uptake was identified.

**Figure 1:**
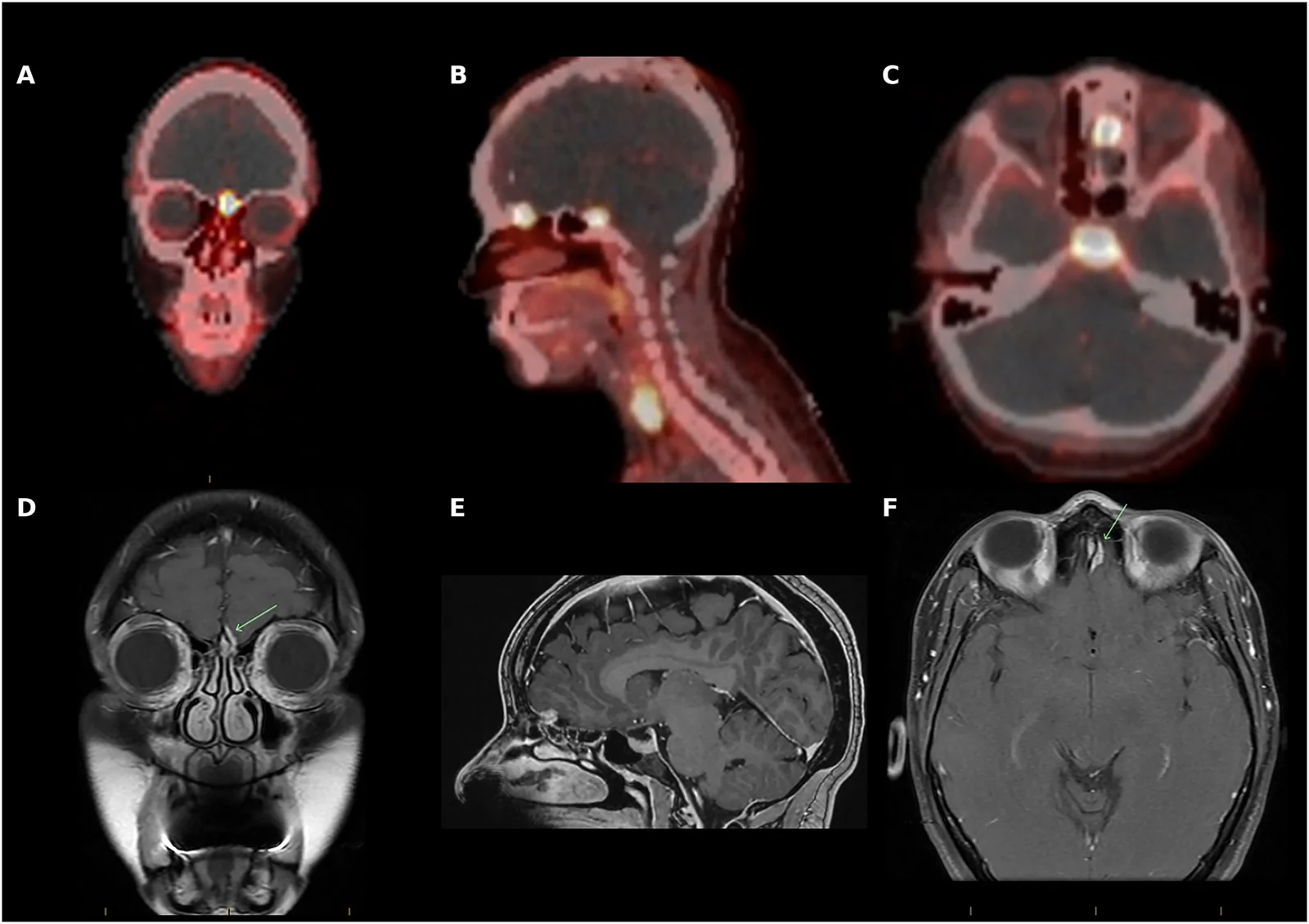
Functional and structural imaging of the olfactory groove lesion. (A-C) Fused ^68^Ga-DOTATATE PET/CT in coronal (A), sagittal (B), and axial (C) planes, demonstrating intense focal radiotracer uptake (SUVmax 19.3) at the anterior cranial base at the junction of the ethmoid air cells and adjacent nasal cavity. (D-F) Contrast-enhanced T1-weighted MRI in coronal (D), sagittal (E), and axial (F) planes; arrows indicate the enhancing dural-based lesion along the left olfactory groove, which corresponds to the focus of uptake on co-registered review. SUV_max_, maximum standardized uptake value.

Because DOTATATE avidity reflects somatostatin receptor 2 (SSTR2) expression rather than a specific histology, the differential for an SSTR2-avid anterior skull base lesion is broad and extends well beyond the classic sites of PMT. In this anatomic region it includes esthesioneuroblastoma (olfactory neuroblastoma), which arises at the cribriform plate and expresses SSTR2 in the majority of cases^2^; paraganglioma, which characteristically overexpresses SSTR2 and is detected by ^68^Ga-DOTATATE with higher sensitivity than by other functional or structural modalities^3^; and metastases from well-differentiated neuroendocrine tumors. Solitary fibrous tumor/hemangiopericytoma and schwannoma enter the radiographic differential of a dural-based mass, although in immunohistochemical series these lesions are typically SSTR2-negative, and SSTR2 expression is comparatively characteristic of meningioma.^4^ Non-neoplastic causes of tracer uptake are also well described, including physiologic uptake in the pituitary and adjacent structures, inflammatory and granulomatous processes, and reactive or healing bone^5^—the last directly relevant here, as it accounts for the mild axial and femoral uptake attributable to this patient’s osteomalacic skeleton and healing insufficiency fractures. Meningiomas themselves strongly express SSTR2 and are characteristically DOTATATE-avid.^6^ Imaging alone therefore could not establish the diagnosis or a causative relationship to TIO, and selective venous sampling for intact FGF-23 was performed.

Selective venous sampling was performed under moderate sedation with midazolam 1 mg and fentanyl 50 mcg intravenously. The right common femoral vein was accessed under real-time ultrasound guidance using a 21-gauge micropuncture set, and a 5F sheath was placed. A 5F headhunter catheter and hydrophilic guidewire were used to selectively catheterize each site, with venography performed at each position to confirm catheter location before sampling. At each site, 3 mL of blood was discarded before a 5 mL specimen was drawn. Samples were obtained in the following order: right internal jugular vein, left internal jugular vein, left subclavian vein, superior vena cava, inferior vena cava, and finally the peripheral specimen from the right common femoral sheath. Total contrast volume was 20 mL of iohexol 300 and fluoroscopy time was 5.8 min. There were no immediate complications.

Concentrations were highest in the jugular venous system, with 131 pg/mL in the left internal jugular vein and 125 pg/mL in the right internal jugular vein, compared with 98 pg/mL in the peripheral (right common femoral) specimen, and declined through the superior vena cava (120 pg/mL) and left subclavian vein (112 pg/mL) to the inferior vena cava (83 pg/mL) (Figure 2). Although the absolute step-up over the peripheral baseline was modest, at 1.34-fold in the left internal jugular vein, the gradient was internally consistent and directional, with both jugular samples exceeding all central and peripheral samples and the inferior vena cava lowest, a pattern consistent with drainage from the anterior cranial fossa and supporting the olfactory groove lesion as the source of FGF-23 secretion. Sampling was interpreted as supportive rather than definitive.

**Figure 2:**
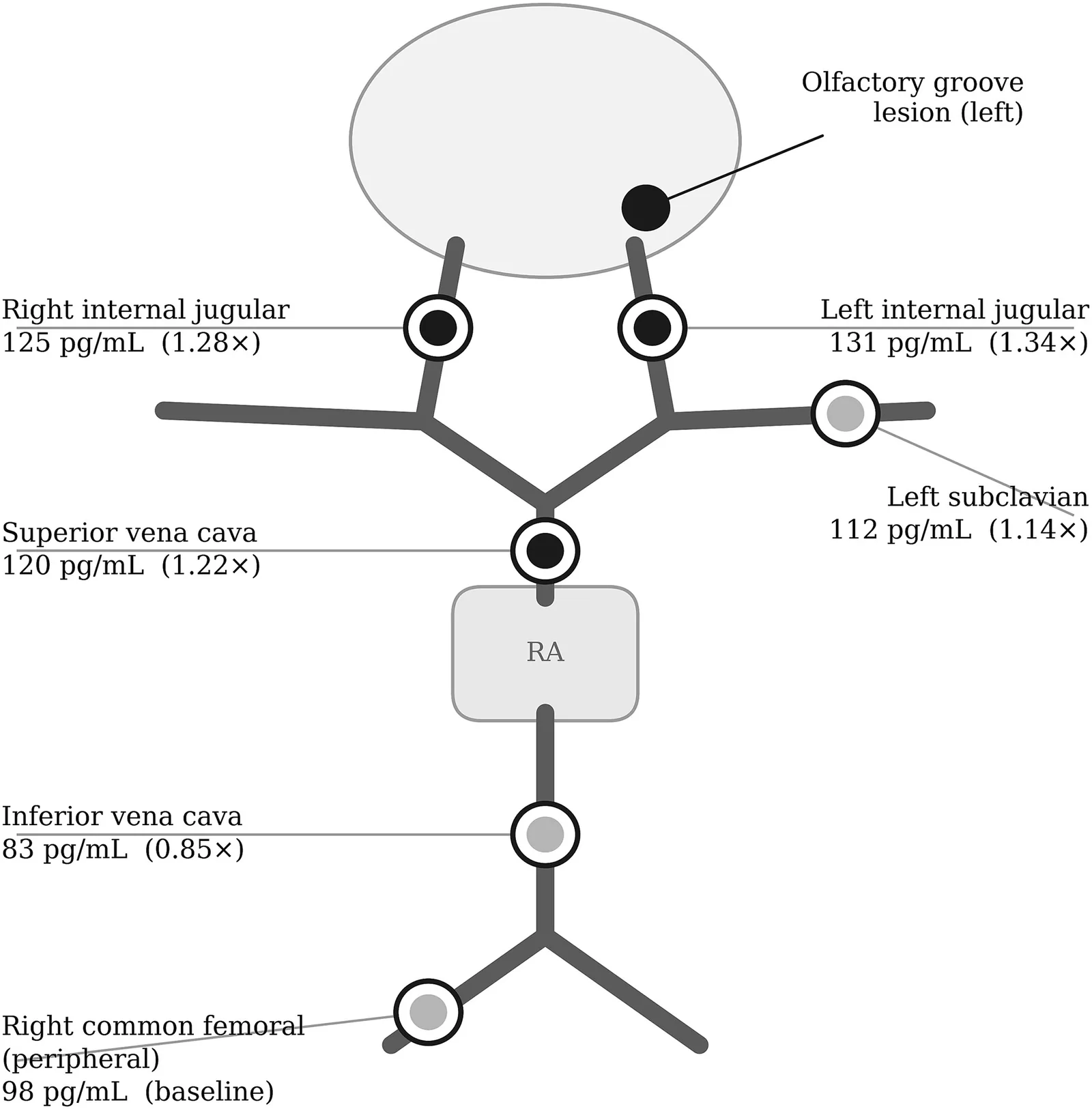
Intact FGF-23 concentrations at selective venous sampling sites. Values are shown at the site sampled, with the ratio to the peripheral (right common femoral) specimen in parentheses. Concentrations were highest in both internal jugular veins and lowest in the inferior vena cava, a pattern consistent with drainage from the anterior cranial fossa. Laterality is shown in anatomical convention, with the patient’s left on the viewer’s right. RA, right atrium.

### Diagnosis

The patient underwent surgical resection of the intracranial lesion through a standard transnasal endoscopic approach to the cribriform plate and frontal skull base. Anterior skull base bone as well as the crista galli were drilled out. The crista galli was hyperostotic, in keeping with expected changes from a meningioma. The tumor itself had the appearance of a meningioma with the normal vascularity and appearance of microcalcifications. It was relatively friable and by the time tumor resection occurred it was largely avascular, again in keeping with a typical meningioma in this location. Gross total resection was achieved.

Intraoperative frozen section interpreted the lesion as a cellular spindle cell neoplasm with a differential including solitary fibrous tumor, meningioma, and PMT, deferring to permanent sections.

Histopathologic evaluation demonstrated a cellular neoplasm with areas of moderate cellularity showing recognizable whorls, syncytial growth, and psammoma bodies, features typically associated with meningioma (Figure 3). The remainder of the tumor showed hypercellularity with hyperchromatic irregular nuclei and scant cytoplasm, without prominent nucleoli, together with sheeting and small-cell change. Scattered mitoses were identified at fewer than 4 per 10 high-power fields, and no necrosis was present. Focal brain parenchymal tissue and nerve were seen surrounding the tumor, without true brain invasion. Classification as World Health Organization grade 2 rested on the presence of 3 minor atypical features—hypercellularity, sheeting, and small-cell change—rather than on mitotic count or brain invasion, neither of which met criteria. The MIB-1 (Ki-67) labeling index was patchily increased to 5%-8%.

**Figure 3:**
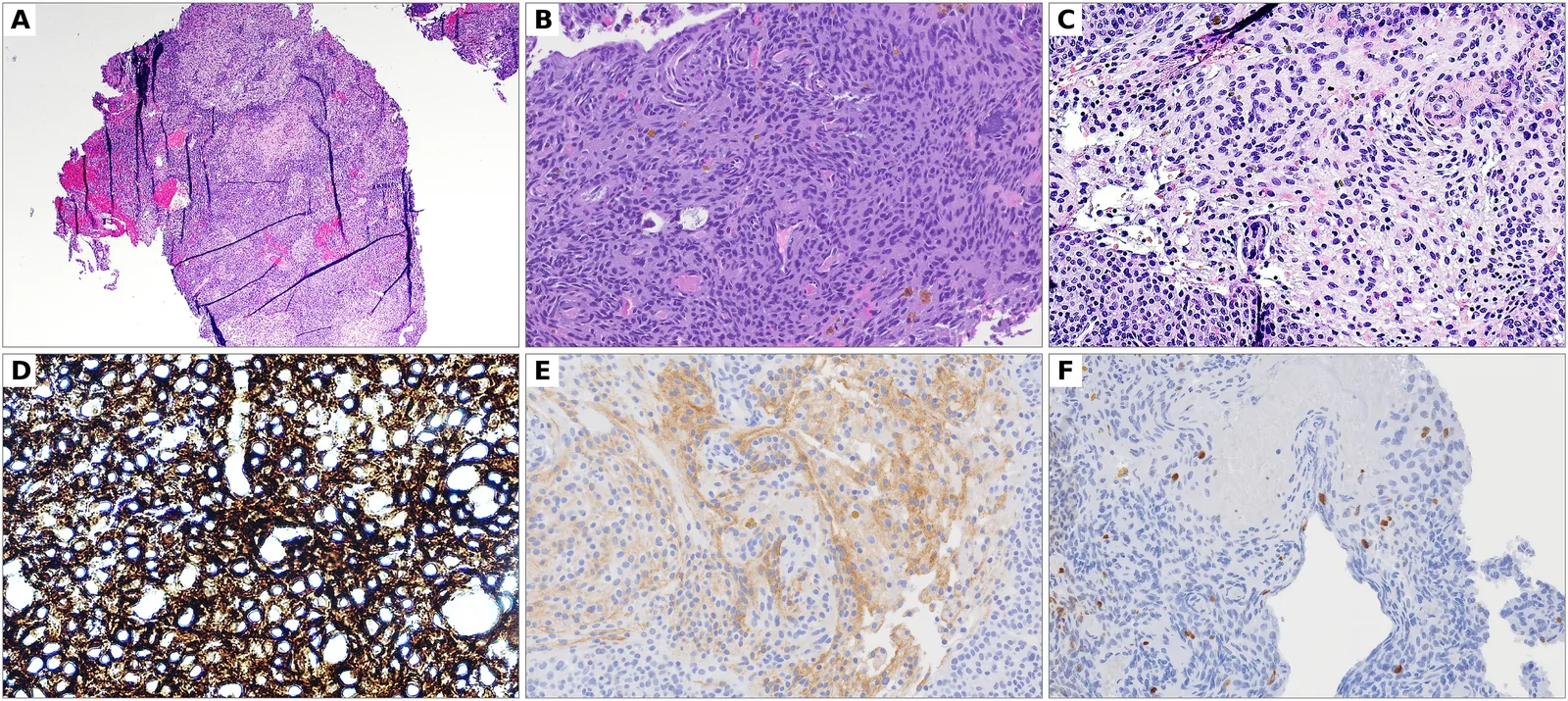
Histopathologic features of the resected lesion. (A) Low-power view showing a cellular neoplasm with variable architecture. (B) Higher-power view demonstrating whorled architecture with syncytial growth and psammoma bodies, features typically associated with meningioma. (C) Hypercellular region with hyperchromatic irregular nuclei, sheeting, and small-cell change. (D) Somatostatin receptor 2A, strongly and diffusely positive. (E) Epithelial membrane antigen, focally positive. (F) MIB-1 (Ki-67), patchily increased to 5%-8%. Hematoxylin and eosin (A-C); immunohistochemistry with hematoxylin counterstain (D-F).

Immunohistochemical analysis demonstrated strong expression of somatostatin receptor 2A and focal epithelial membrane antigen (EMA) positivity. Glial fibrillary acidic protein highlighted the surrounding brain tissue and confirmed the absence of brain invasion. STAT6 was negative, excluding solitary fibrous tumor. Expanded immunohistochemistry showed rare cells positive for smooth muscle actin and focal staining for cluster of differentiation 99 (CD99) in the less cellular areas, with retained integrase interactor 1 nuclear expression, no histone H3 lysine 27 trimethylation nuclear loss, and a wild-type p53 staining pattern p53 staining pattern. Desmin, myogenin, CD68, nuclear protein in testis, CD34, Wilms tumor 1, NK2 homeobox 2, mucin 4, synaptophysin, S100, estrogen receptor, and pan-cytokeratin (AE1/AE3) were negative.

FGF-23 in situ hybridization performed at Mayo Clinic Laboratories was positive.

Because of the atypical morphologic and clinical context, expanded ancillary testing was performed. Next-generation sequencing did not identify canonical meningioma-associated alterations. Chromosomal microarray demonstrated a large deletion on chromosome 12q, multiple deletions on chromosome 13q without involvement of RB1, and gain of chromosome 17, without NF2 loss or typical meningioma-associated 1p, 14q, or 10q abnormalities. DNA methylation profiling performed at the National Institutes of Health classified the lesion as a mesenchymal neoplasm, not elsewhere classified, with the highest similarity to meningioma but without a definitive match; the analysis was fusion negative.

The final surgical pathology diagnosis was mesenchymal tumor, favoring PMT over meningioma. Taken together with biochemically proven FGF-23–mediated phosphate wasting, venous sampling localizing hormone secretion to the anterior cranial fossa, and immediate postoperative biochemical cure, the integrated clinicopathologic diagnosis is an intracranial PMT with overlapping meningioma-like morphology.

### Treatment and progression

The patient had received no treatment for hypophosphatemia or metabolic bone disease before referral, and all diagnostic urine and serum studies were obtained before any supplementation was started. Once FGF-23–mediated phosphate wasting was confirmed, and while localization and surgical planning were under way, she was treated with ergocalciferol 50 000 IU weekly, calcitriol 0.5 mcg 3 times daily, calcium citrate 500 mg twice daily, and neutral sodium and potassium phosphate 250 mg 4 times daily.

Serum intact FGF-23 had fallen below the assay detection limit (<14 pg/mL) by the first postoperative morning, consistent with complete removal of the FGF-23–secreting tumor. Serum phosphorus rose from 1.2 mg/dL preoperatively to a first sustained normal value of 3.0 mg/dL on postoperative day 5, with a transient decline to 1.4 mg/dL on the evening of the first postoperative day reflecting rapid skeletal remineralization (Table 1). Correction was concordant across both assay methods: C-terminal FGF-23 fell from 284 RU/mL preoperatively to 123 RU/mL, within the reference range, by postoperative day 10, and intact FGF-23 was 18 pg/mL at 10.5 mo. Serum 1,25-dihydroxyvitamin D rose markedly after resection, from 29 pg/mL preoperatively to 218 pg/mL at 1 mo, and remained above the reference range at 129 and 142 pg/mL at 4 and 10.5 mo. This rebound reflects release of renal 1α-hydroxylase from prolonged FGF-23–mediated suppression, compounded by the persisting secondary hyperparathyroidism, and it resolved without intervention as calcitriol supplementation was withdrawn.

Clinically, the patient reported marked improvement in bone pain and muscle weakness. Phosphate, calcitriol, and calcium supplementation were discontinued over subsequent weeks. Ergocalciferol 50 000 IU weekly was continued for 6 wk to complete correction of the baseline 25OHD deficit, then changed to cholecalciferol 4000 IU daily and subsequently reduced to a maintenance dose of 2000 IU daily. The patient regained independent ambulation and demonstrated progressive musculoskeletal recovery.

On follow-up DXA 14 mo postoperatively, LS (L1-L4) BMD had increased from 0.98 to 1.094 g/cm^2^, a gain of approximately 12%, with the Z-score improving from −2.1 to +0.4. Trabecular bone score was not obtained. Hip densitometry was not repeated because bilateral femoral internal-fixation hardware precludes valid measurement, consistent with International Society for Clinical Densitometry positions. Total alkaline phosphatase fell from a peak of 235 U/L at presentation (reference range, 31-125 U/L) to 149 U/L at 1 mo and 72 U/L at 17 mo postoperatively, and bone-specific alkaline phosphatase fell from 61.3 to 43.3 μg/L over the first month. Tubular reabsorption of phosphate rose from 85.4% to 95.8%. No new fractures occurred during follow-up, and no adverse or unanticipated postoperative events were observed.

## Discussion

Tumor-induced osteomalacia is an acquired paraneoplastic syndrome driven by excess tumoral FGF-23, which reduces proximal tubular phosphate reabsorption and suppresses 1,25-dihydroxyvitamin D synthesis, producing chronic hypophosphatemia and defective skeletal mineralization.^7^^,^^8^ Patients present with progressive bone pain, proximal weakness, and insufficiency fractures, often after prolonged diagnostic delay, because the responsible lesions—usually PMTs—are small and anatomically diverse.^8–11^ Somatostatin receptor-based functional imaging is now central to localization, since many PMTs express SSTR2 and are detectable with ^68^Ga-labeled tracers,^12^ and selective venous sampling for intact FGF-23 provides confirmation once at least one candidate lesion has been identified.^13^^,^^14^

Intracranial PMTs are exceedingly rare but increasingly recognized. In a systematic review of 29 reported cases, there was a marked female predominance (22 of 29, 76%), osteomalacia was present in 86% and bone fractures in 34%, and the anterior cranial fossa was the most common site of involvement (14 of 29, 48%)—the region where dura, skull base, and craniofacial mesenchyme intersect; most previously reported meningioma-like lesions associated with TIO have ultimately been classified as PMT or another mesenchymal tumor rather than pure meningioma.^15^ The central pitfall is that small dural-based enhancing lesions are routinely read as meningiomas, the most common dural tumor and a frequent incidental finding managed conservatively,^16^ yet many entities mimic meningioma radiographically,^16^ and “dural-based” is a description, not a diagnosis. Because meningiomas strongly express SSTR2 and are characteristically DOTATATE-avid,^6^ intense uptake identifies an SSTR2-avid lesion requiring endocrine–radiologic–pathologic correlation, not a PMT per se. In a patient with biochemically confirmed FGF-23–mediated phosphate wasting, presuming such a lesion incidental risks delaying curative resection.^11^ Direct venous sampling helps resolve this ambiguity by indicating whether a meningioma-appearing lesion is causative or incidental. In the largest published series, a peripheral-to-tumor-bed ratio of at least 1.6 was diagnostic^13^; our maximal jugular ratio of 1.34 falls below that threshold, and sampling was therefore interpreted as supportive rather than definitive. The decisive evidence for causality was the fall in intact FGF-23 below the assay detection limit by the first postoperative morning, with sustained normophosphatemia and normalization of tubular phosphate reabsorption thereafter.

The pathologic diagnosis proved less straightforward than the endocrine one. The initial read was atypical meningioma, based on a classic olfactory-groove appearance directly comparable to numerous olfactory-groove meningiomas from our high-volume skull base program. Recognizable whorls, syncytial growth, and psammoma bodies coexisted with hypercellular regions showing hyperchromatic irregular nuclei, sheeting, and small-cell change. The tumor was focally EMA-positive and strongly SSTR2-positive, with no canonical meningioma-associated alterations on next-generation sequencing and a methylation profile falling closest to meningioma without a definitive match. Positive FGF-23 in situ hybridization, together with the absence of canonical meningioma alterations, ultimately favored PMT. The differential had until then spanned FGF-23–secreting meningioma, meningioma with PMT inclusion, and PMT with meningioma-like features—a distinction that did not alter management, but which required multiple ancillary studies to resolve.

One explanation for the meningioma-mimicking architecture is that the skull–dura interface itself provides a mesenchymal substrate for PMT development: the cranial meninges harbor mesenchymal derivatives of multiple embryonic lineages and interact with the developing calvaria, where meningeal signaling is required for normal osteogenesis,^17^^,^^18^ and FGF-23 is a native program of bone-lineage cells that could be aberrantly retained after transformation of an osteogenic-adjacent population.^19^

These observations argue against overreliance on any single marker. Gross morphology is nonspecific, and SSTR2 positivity does not discriminate PMT from meningioma; the relevant entities overlap substantially (Table 2), and diagnosis is better served by integrating all available data than by privileging an isolated finding. The practical lesson is that in a patient with confirmed FGF-23–mediated phosphate wasting, even a small dural-based lesion should be treated as a high-priority diagnostic target rather than presumed incidental, and that accurate diagnosis requires synthesis of endocrine physiology, functional imaging, pathology, and molecular classification. We therefore advocate an aggressive, multimodal search for the FGF-23 source directed toward resection, together with further research into FGF-23 production across diverse mesenchymal tissues.

**Table 2:** Comparison of meningioma, meningioma harboring/mimicking PMT, and PMT, with findings in the present case.

**Feature**	**Meningioma**	**Meningioma harboring/mimicking PMT**	**PMT (PMTMCT)**	**Present case**
**Cell of origin**	Arachnoid cap (meningothelial) cells	Meningothelial component plus coexistent phosphaturic mesenchymal cells	Primitive mesenchymal/fibroblast-like cells	Indeterminate; mesenchymal
**Location**	Any dural surface	Skull base/anterior cranial fossa	Bone and soft tissue; intracranial rare, anterior cranial fossa	Left olfactory groove, anterior cranial fossa
**Histology**	Whorls, psammoma bodies, syncytial cells	Meningothelial features with bland spindle cells, grungy calcified matrix	Bland spindle/stellate cells, flocculent calcification, HPC-like vasculature, giant cells	Whorls, syncytial growth and psammoma bodies with hypercellularity, sheeting and small-cell change
**Mitoses/grade**	Variable by grade	Variable	Usually low	<4 per 10 HPF; no necrosis; no brain invasion; WHO grade 2 on three minor atypical features
**MIB-1 (Ki-67)**	Variable by grade	Variable	Usually low	Patchily increased, 5%-8%
**IHC: SSTR2A**	Positive (strong)	Positive	Variable/positive	Strongly positive
**IHC: EMA**	Positive	Positive (meningothelial areas)	Negative/focal	Focally positive
**IHC: GFAP**	Negative	Negative	Negative	Negative in tumor (highlights adjacent brain)
**IHC: STAT6**	Negative	Negative	Negative	Negative
**IHC: ER/PR**	PR often positive	Variable	Negative	ER negative
**Molecular**	NF2 loss, TRAF7, AKT1, SMO, KLF4	Meningioma alterations ± FGFR1 events	FN1-FGFR1 or FN1-FGF1 fusion; KL rearrangement	No canonical meningioma alterations on NGS; 12q deletion, 13q deletions, chromosome 17 gain; methylation—mesenchymal neoplasm NEC, fusion negative
**FGF-23 expression**	Negative	Positive (PMT component)	Positive—drives TIO	Positive
**DOTATATE avidity**	Avid	Avid	Avid (overlap)	Avid; SUVmax 19.3
**Management**	Resection/RT per WHO grade	Resection; phosphate normalization confirms PMT	GTR curative; if unresectable, burosumab or phosphate/calcitriol	Endoscopic resection; biochemical cure

SSTR2A positivity and DOTATATE avidity are shared across all 3 entities and cannot distinguish them; FGF-23 in situ hybridization and the FN1-FGFR1/FN1-FGF1 fusion are the discriminating features. In the present case, meningioma-like morphology and strong SSTR2A positivity coexisted with absent canonical meningioma alterations, a fusion-negative mesenchymal methylation class, and positive FGF-23 in situ hybridization, and the final surgical pathology diagnosis favored phosphaturic mesenchymal tumor over meningioma. Abbreviations: EMA, epithelial membrane antigen; ER, estrogen receptor; GFAP, glial fibrillary acidic protein; GTR, gross total resection; HPC, hemangiopericytoma; HPF, high-power field; NEC, not elsewhere classified; NGS, next-generation sequencing; PMT, phosphaturic mesenchymal tumor; PMTMCT, PMT mixed connective tissue type; PR, progesterone receptor; RT, radiotherapy; SSTR2A, somatostatin receptor 2A; SUV_max_, maximum standardized uptake value.

### Unanswered questions

Several questions remain regarding the biology of intracranial phosphaturic tumors. The present case was fusion negative on methylation-based analysis, raising the question of whether meningeal phosphaturic tumors share the fusion architecture described in PMTs arising elsewhere^20^ or instead represent a distinct meningeal subset. Which specific cell population at the skull–dura interface expresses FGF-23 in these tumors? Can spatial or single-cell profiling distinguish tumor-cell FGF-23 production from stromal-microenvironment expression? Finally, can methylation-based classification define whether intracranial phosphaturic tumors cluster with known mesenchymal tumor classes or represent a unique epigenetic category?

## Data Availability

All data generated or analyzed during this case report are included in this published article.

## Permission to reproduce material from other sources

Not applicable; no third-party material is reproduced.
